# Are fecal samples an appropriate proxy for amphibian intestinal microbiota?

**DOI:** 10.1002/ece3.10862

**Published:** 2024-01-31

**Authors:** Ivan P. Y. Lam, Jonathan J. Fong

**Affiliations:** ^1^ School of Biological Science The University of Hong Kong Hong Kong China; ^2^ Science Unit Lingnan University Hong Kong China

**Keywords:** amphibian microbiome, fecal microbiota, gut microbiota, non‐invasive sampling, wildlife conservation

## Abstract

The intestinal microbiota, an invisible organ supporting a host's survival, has essential roles in metabolism, immunity, growth, and development. Since intestinal microbiota influences a host's biology, application of such data to wildlife conservation has gained interest. There are standard protocols for studying the human intestinal microbiota, but no equivalent for wildlife. A major challenge is sampling the intestinal microbiota in an effective, unbiased way. Fecal samples are a popular proxy for intestinal microbiota because collection is non‐invasive and allows for longitudinal sampling. Yet it is unclear whether the fecal microbiota is representative of the intestinal microbiota. In wildlife studies, research on the sampling methodology is limited. In this study focusing on amphibians, we characterize and compare the microbiota (small intestine, large intestine, and feces) of two Hong Kong stream‐dwelling frog species: Lesser Spiny Frog (*Quasipaa exilispinosa*) and Hong Kong Cascade Frog (*Amolops hongkongensis*). We found that the microbiota of both species are similar at the level of phylum and family, but diverge at the level of genus. When we assessed the performance of fecal microbiota in representing the intestinal microbiota in these two species, we found that (1) the microbiota of the small and large intestine differs significantly, (2) feces are not an appropriate proxy of either intestinal sections, and (3) a set of microbial taxa significantly differs between sample types. Our findings raise caution equating fecal and intestinal microbiota in stream‐dwelling frogs. Sampling feces can avoid sacrifice of an animal, but researchers should avoid over‐extrapolation and interpret results carefully.

## Graphical abstract

This study compares microbiota of feces, small, and large intestine in stream‐dwelling amphibians. We find that microbiota differ significantly between sample types. Therefore, we caution the use of fecal microbiota as the proxy of intestinal microbiota in future studies.

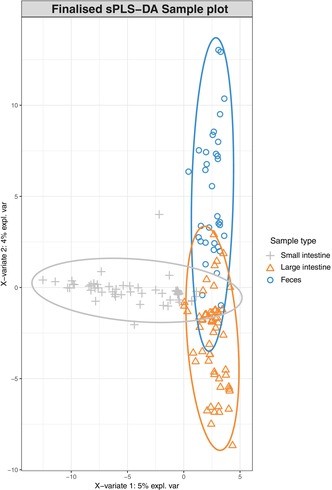

## 1 INTRODUCTION

A functional gastrointestinal system is not only supported by healthy organs but also a balance of associated microbiota. The intestinal microbiota is a group of microorganisms (bacteria, fungi, archaea, protozoa, and viruses) that establishes a dynamic, microscopic ecosystem tolerated by a host's immune system (Hooper et al., 2012). As the number of symbiotic microorganisms is similar to the number of cells of the host (Sender et al., 2016), the microbiota can be regarded as a virtual organ that supports a host's health. Research has highlighted the essential roles of the gut microbiota, such as nutrient absorption (Krajmalnik‐Brown et al., 2012), energy metabolism (Cani & Delzenne, 2009; Heiss & Olofsson, 2018), immunity (Belkaid & Hand, 2014), detoxification (Claus et al., 2016; Diaz‐Bone & Van de Wiele, 2010), and mental health (Butler et al., 2019; Clapp et al., 2017; Ganci et al., 2019). With these important roles, researchers have explored applying gut microbiota research to wildlife conservation; microbiota studies have provided a new insight, such as linking changes of the intestinal microbiota in endangered species to anthropogenic factors (Trevelline et al., 2019).

The first step in studying the intestinal microbiota is sampling. An ideal sampling method is non‐invasive, has low risk of sample contamination, has low risk of procedure bias, allows longitudinal sampling, and allows multiple site sampling (Tong et al., 2020). In wildlife studies, commonly taken samples are non‐invasive feces (Alfano et al., 2015; Amato et al., 2013; Ingala et al., 2018; Menke et al., 2015) and minimally invasive rectal swabs (Alfano et al., 2015; Artim et al., 2019; Zhou, Nelson, et al., 2020). Although both sample types allow for efficient sampling, they only function as a proxy, and it is unclear how well they represent the intestinal microbiota.

Recent studies on a variety of taxa assessed the effectiveness of using feces and rectal swabs to study the intestinal microbiota, and the results were mixed. For bats, Ingala et al. (2018) found that the diversity and composition of microbiota significantly differed between samples of guano (feces) and the distal intestine (last section of large intestine). For terrestrial amphibians, Zhou, Nelson, et al. (2020) compared the microbiota of feces, rectal swabs, small intestine, and large intestine and found only the rectal swab microbiota was representative of the large intestine microbiota. Furthermore, comparison in lizards cautions the use of feces as a proxy of any intestinal sections (Bunker et al., 2021). In birds, Videvall et al. (2018) found that the fecal microbiota reflected that of the colon microbiota, but neither the ileum (last part of the small intestine) nor caecum (first part of the large intestine). Berlow et al. (2019) found a similar result where the fecal microbiota was distinct compared to the small intestine, but indistinguishable from large intestinal microbiota. On the contrary, a study of the domestic chicken found that fecal samples performed well in representing dominant taxa in the small intestine (the duodenum, jejunum, and ileum) and large intestine (cecum), thus having potential in reflecting community structure when interpreted cautiously (Yan et al., 2019). Since the structure of the intestinal tract varies between different taxa (Karasov & Douglas, 2013), it is expected that studies of different taxa find different results. These mixed results suggest that generalization is difficult across study systems in terms of taxon and habitat.

The intestinal tract is a complex ecosystem that drastically differs across sections in terms of chemistry, physical structure, enzymatic activities, and immune system activity. As a result, a distinct microbiota inhabits each intestinal section, a phenomenon well documented in humans (Donaldson et al., 2016; Thursby & Juge, 2017; Vaga et al., 2020), other mammals (Anders et al., 2021; Gu et al., 2013; Kohl et al., 2018; Yang et al., 2020), fish (Zhu et al., 2021), birds (Choi et al., 2014), and reptiles (Kohl et al., 2017). Since the microbiota is heterogeneous along the intestinal tract, it is impossible for feces to mirror the microbiota of each section. So the question shifts to “how much overlap is there between feces and small/large intestine?” and “is the feces microbiota more similar to small or large intestine?”

In this study, we assess the suitability of using feces to study the intestinal microbiota of stream‐dwelling amphibians. We first characterize and compare microbiota (the small intestine, large intestine, and feces) of two Hong Kong frog species: Lesser Spiny Frog (*Quasipaa exilispinosa*) and Hong Kong Cascade Frog (*Amolops hongkongensis*). Next, in these two species, we assess the overlap between the microbiota of the small intestine, large intestine, and feces. We use a compositional analysis approach since it reduces bias in microbiota studies (Calle, 2019; Gloor et al., 2017; McMurdie & Holmes, 2014). Results from this study will advance research by evaluating a common sampling protocol specifically for stream‐dwelling frogs and contribute to our general understanding for wildlife studies; verifying the usefulness of feces would minimize the need to sacrifice animals and facilitate the study of gut microbiota, especially in endangered species, while an opposite result would alert researchers to develop new methods to study the gut microbiota and/or cautiously interpret data from fecal samples.

## 2 MATERIALS AND METHODS

### 2.1 Study species

**FIGURE 1:**
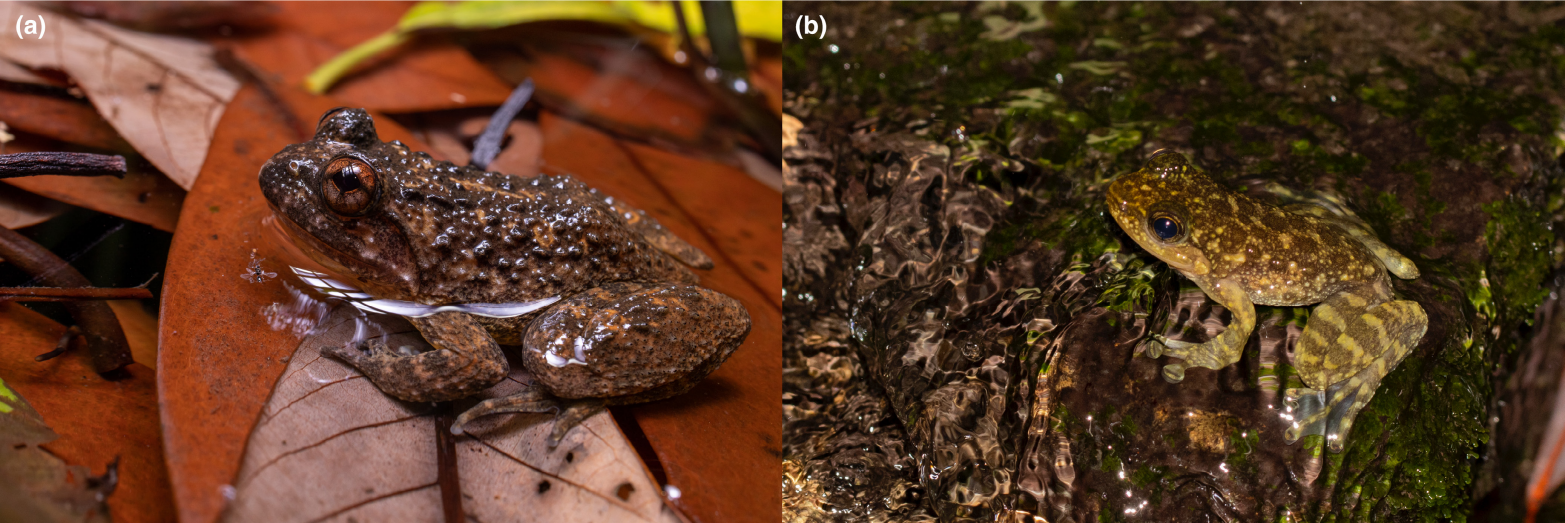
Two stream‐dwelling frogs chosen as the study species. (a) Lesser Spiny Frog (*Quasipaa exilispinosa*) and (b) Hong Kong Cascade Frog (*Amolops hongkongensis*).

Two stream‐dwelling species were chosen as the study species: Lesser Spiny Frog (*Q. exilispinosa*) and Hong Kong Cascade Frog (*A. hongkongensis)* (Figure 1). *Quasipaa exilispinosa* has a wide geographical distribution, being found throughout southern China in Hunan, Fujian, Guangxi, and Guangdong Provinces, including Hong Kong (IUCN SSC Amphibian Specialist Group, 2020; Karsen et al., 1998). *Amolops hongkongensis* was once thought to be endemic to Hong Kong but is also found in Guangdong Province, China (IUCN SSC Amphibian Specialist Group, 2021; Karsen et al., 1998). These two species are of conservation concern since they are affected by habitat loss and/or hunting; *Quasipaa exilispinosa* is currently listed as “Least Concern” on the IUCN Red List of Threatened Species, but previously was “Vulnerable”, while *A. hongkongensis* is currently listed as “Endangered” (IUCN SSC Amphibian Specialist Group, 2020, 2021). Hong Kong houses healthy populations (wide distribution; high population density), which provides a unique opportunity to collect baseline data applicable to conservation. Permits to collect these two species were issued by the Hong Kong Government [Permit # (112) in AF GR CON 09/51 Pt.7], and all methods were approved by the Hong Kong Government [Permit # (20‐51) in DH/HT&A/8/2/8 Pt.1] and Lingnan University Ethics Committee [EC050/2021].

### 2.2 Study site

The study was conducted in the Hong Kong Special Administrative Region, China (22°09′–22°37′ N, 113°50′–114°30′ E). Hong Kong has a subtropical climate characterized by a cool dry season (November to February) and a hot, humid wet season (May to August) separated by mild autumns and springs (Dudgeon & Corlett, 2011). Microbiota sampling (see below) was conducted during the wet season of 2021. Sampling sites were chosen because they (1) have high individual abundance to collect enough samples for a robust study without harming the population; (2) consist of natural, protected environments to minimize confounding anthropogenic factors on microbiota; and (3) are easy access for efficient sampling and reduce transport time for better sample preservation. Seven sites were chosen that match these criteria: (1) Tai Mo Shan Country Park (Sze Lok Yuen), Ma On Shan Country Park ([2] Mui Tsz Lam and [3] Tai Shui Hang), (4) Lion Rock Country Park, (5) Tai Tam Country Park, (6) Lantau Country Park (Tei Tong Tsai), and (7) Tai Lam Country Park (Ting Kau).

### 2.3 Microbiome sampling

A total of 60 individuals (29 *Q. exilispinosa*; 31 *A. hongkongensis*) were captured. A maximum of four types of microbiota samples were collected from each individual—skin (for another study). small intestine, large intestine, and if available, feces. Details are as follows.

### 2.4 Frog surveys

Night surveys were conducted within 3 h after sunset (typically 18:00–22:00), from June to August 2021. Each survey included 3–4 surveyors experienced in herpetological field work. Surveyors walked along the stream looking for the two target species using headlamps and flashlights. Individuals were captured with gloved hands, with gloves changed between individuals to prevent cross‐contamination. After sampling skin microbiota for another study, each individual was stored in separate air‐filled plastic bags for transport to laboratory within 2 h.

### 2.5 Fecal microbiota

All equipment was sterilized with a 10% bleach solution beforehand to avoid contamination. Upon arrival to the laboratory, each individual was kept separately in sterile plastic boxes for 12–24 h waiting for defecation. The boxes have a wire mesh floor that allows the feces to fall and avoid contamination by the individual. Each box was humidified by spraying sterile water and kept in dark to minimize disturbance. Approximately 9 h after capture, boxes were checked hourly for defecation. No food was provided during the process. Fecal samples were collected using sterile forceps and stored in tubes at −80 °C until DNA extraction. Individuals were euthanized for sampling intestinal microbiota (see below) after defecation or after 24 h without defecation.

### 2.6 Intestinal microbiota

Each individual was euthanized by overdosing with ethyl 3‐aminobenzoate methanesulfonate in the laboratory (MS222; Sigma‐Aldrich, St. Louis, MO, USA) (American Veterinary Medical Association, 2020). The entire intestine was dissected from the end of the stomach to the cloaca. The small and large intestine were identified morphologically. A 2‐cm piece of the anterior and posterior end of the intestine was taken as the small and large intestine, respectively (Tong et al., 2014). We could not identify intestinal subsections morphologically (e.g., duodenum, jejunum, and ileum of the small intestine), so this information was excluded from our analyses. Each segment was rinsed to remove transient bacteria and intestinal content with 2 mL sterile water using a pipette. Each segment was then cut longitudinally on one side, laid flat, and the gut microbiota was collected by swabbing using an ESwab™ (Copan Diagnostic Inc., CA, USA). The swab was drawn across the inner surface of the intestine segment 10 times (1 time = forward and backward). The swabs were stored at −80 °C until DNA extraction. For a negative control, two types of blank swabs (opened during dissection and DNA extraction) were included to account for potential contamination from the environment during those respective laboratory steps. To extend the utility of these specimens, frog bodies were preserved and accessioned into the herpetology specimen collection of the Lingnan University Natural History Collection.

### 2.7 DNA extraction

DNA extraction was conducted at the laboratory of Lingnan University in a biological safety cabinet (Airstream Class II S series). DNA was extracted from swabs using the QIAamp®BiOstic® Bacteremia DNA Kit (Qiagen GmbH, Hilden, Germany) following amendments verified by the manufacturer. The amendments are summarized as follows—in the first step, 450 μL of MBL buffer was added to each PowerBead tube: (1) swabs were defrosted and then placed in PowerBead tubes, (2) feces were subsampled (250 mg) and placed in PowerBead tubes, and (3) the subsequent steps followed the manufacturer's protocol starting from “vortex for 10 s and incubation at 70 °C for 15 min”. A blank sample was included for every batch of DNA extraction as a negative control. DNA extractions were quantified by Qubit™ 3 Fluorometer with the dsDNA BR Assay Kit (Thermo Fisher Scientific Inc., MA, USA).

### 2.8 Library preparation and sequencing

Sequencing libraries were prepared according to the Illumina protocol (“16S Metagenomic Sequencing Library Preparation Part #15044223 Rev. B protocol”). The V4 region of the 16S rRNA gene was amplified by PCR using The Earth Microbiome Project V4 primer pair coupled with Illumina adapter overhang sequences (515F [Parada]: 5′‐TCGTCGGCAGCGTCAGATGTGTATAAGAGACAGGTGYCAGCMGCCGCGGTAA‐3′, 806R [Apprill]: 5′‐GTCTCGTGGGCTCGGAGATGTGTATAAGAGACAGGGACTACNVGGGTWTCTAAT‐3′; Apprill et al., 2015; Caporaso et al., 2011; Parada et al., 2016). For each reaction, 2 ng of DNA was amplified with 5× reaction buffer, 1 mM of dNTP mix, 500 nM of each PCR primer, and Herculase II fusion DNA polymerase (Agilent Technologies, Santa Clara, CA). Thermal cycling conditions were as follows: 3 min at 95 °C; 25 cycles of 30 s at 95 °C, 30 s at 55 °C, and 30 s at 72 °C; followed by 5 min at 72 °C. The first PCR product was purified with AMPure beads (Agencourt Bioscience, Beverly, MA). For each sample, 2 μL of the first PCR product was amplified for library construction using a unique Nextera XT Index Primer (Illumina, Inc., San Diego, CA). Thermal cycling conditions were the same as the first PCR, but with only 10 cycles. The 2nd PCR product was then purified using AMPure beads (Agencourt Bioscience) and quantified using two approaches: (1) qPCR according to the qPCR Quantification Protocol Guide (KAPA Library Quantification kit for Illumina Sequencing platforms) and (2) TapeStation system and D1000 ScreenTape (Agilent Technologies, Waldbronn, Germany). The pooled products were paired‐end sequenced with the Herculase II Fusion DNA Polymerase Nextera XT Index V2 Kit on an Illumina MiSeq PE300 (Illumina, Inc.). A negative control was included in each lane to test for contamination. All library preparation and sequencing work was conducted at Macrogen (Seoul, South Korea).

### 2.9 Raw data processing

Bioinformatics work was performed using Quantitative Insights into Microbial Ecology 2 (QIIME 2, version 2022.11) (Bolyen et al., 2019) and R (R Core Team, 2021). Raw sequence data were demultiplexed by Macrogen (Seoul, South Korea). We evaluated the quality of demultiplexed reads and trimmed to remove poor‐quality bases. Sequence quality control and denoising were performed with DADA2 (Callahan et al., 2016) to generate amplicon sequence variants (ASVs) using q2‐dada2. Representative sequences were aligned with MAFFT (Katoh & Standley, 2013) using q2‐alignment. To maximize taxonomic classification accuracy, we assigned taxonomy to ASVs using a weighted Bayes classifier that incorporates information of environment‐specific taxonomic abundance (Kaehler et al., 2019), pre‐trained on the 16S 515F/806R region in SILVA 138 (Quast et al., 2013) (MD5: b9476399080d189b4c9917d1246e7c69) (Bokulich et al., 2018; Robeson II et al., 2021) using the q2‐feature‐classifier (Bokulich et al., 2018). Sequences were removed if unassigned at the domain level or assigned to be mitochondria/chloroplast/archaea. The format of the metadata file used in QIIME 2 was validated by the cloud‐based Google Sheets add‐on Keemei (Rideout et al., 2016). The output QIIME 2 files were exported to R for subsequent analysis using the R package “qiime2R” (Bisanz, 2018).

### 2.10 Prefiltering

To improve microbiota profiling and avoid spurious results (Bokulich et al., 2013; Huse et al., 2010; Kunin et al., 2010), we adopted a strict prefiltering approach following Lê Cao et al. (2016); we removed samples with less than 10 ASV counts and ASVs with proportional counts across all samples below 0.01%.

### 2.11 Statistical analyses

Our statistical analyses involved three steps to compare sample types (feces, small intestine, and large intestine): (1) examine alpha diversity using taxa bar plots and Shannon diversity estimates (Willis & Bunge, 2015; Willis & Martin, 2022), (2) perform multivariate differential abundance analysis using the mixMC framework (Lê Cao et al., 2011, 2016), and (3) perform univariate differential abundance analysis using ANOVA‐like Differential Gene Expression Analysis (ALDEx2) (Fernandes et al., 2013, 2014).

### 2.12 Alpha diversity

To characterize microbiota samples from both invasive (small intestine and large intestine) and non‐invasive (feces) sampling methods, microbiota was analyzed at three taxonomic levels (phylum, family, and genus) for each species separately. The top 20 most abundant taxa were identified and shown in bar plots created using QIIME 2 plugin “q2‐taxa‐barplot” (Bolyen et al., 2019) and R package “ggplot2” (Wickham, 2016). We compare the microbiota of the two species using Venn diagrams created by the R package “VennDiagram” (Chen & Boutros, 2011).

Shannon diversity estimates allow prediction of unobserved species counts in samples to estimate the total diversity (Willis & Bunge, 2015; Willis & Martin, 2022). Estimates for each sample are equipped with upper and lower errors to account for uncertainty. To calculate Shannon diversity estimates at the ASV level, we use the R function *divnet()* in the R package “DivNet” (Willis & Martin, 2022) integrated with “phyloseq” (McMurdie & Holmes, 2013). To test the difference of Shannon diversity estimates between sample types at the 95% confidence level, we used the R function *betta_random()* in the R package “breakaway” (Willis et al., 2017; Willis & Bunge, 2015). In the model, we included six variables as fixed factors: sample type, species, snout‐vent length, body weight, and sampling location; and individual identity as the random factor. The best model was selected by the lowest Akaike's Information Criterion with correction (AICc) value. Subsequently, to perform pairwise comparison between sample types, we used the R function *betta_lincom()* from the same R package. To illustrate the difference of Shannon diversity estimates, box‐and‐whisker diagrams and scatter plots were created using the R package “ggplot2” (Wickham, 2016) when appropriate. A preliminary analysis was conducted separately on datasets of each species and then repeated with a combined dataset if similar results were obtained in the preliminary analysis.

### 2.13 Multivariate differential abundance analysis

To test whether the microbiota differs between sample types (feces, small intestine, and large intestine), the mixMC framework was used using the R package “mixOmics” (Lê Cao et al., 2011, 2016; Rohart et al., 2017). The mixMC framework employs sparse partial least squares discriminant analysis (sPLA‐DA) to detect subtle between‐group differences in microbiota by accounting for the sparse compositional nature and handling high inter‐subject variability (Lê Cao et al., 2011, 2016). A preliminary analysis was conducted separately on datasets of each species and then repeated with a combined dataset if a similar result was obtained in the preliminary analysis.

An exploratory PCA was performed to observe the general structure and clustering of the dataset. To reduce spurious results, we performed a centered log ratio transformation prior to analysis. To determine the number of principal components best discriminating sample types, the elbow method was applied to a bar plot generated by the R function *plot()* on a PCA object with 10 components. Next, an initial sPLS‐DA model was generated using R function *plsda()* in the R package “mixOmics” (Rohart et al., 2017). To improve the model, the number of components (the “ncomp” parameter) and the number of variables (the “keepX” parameter) were optimized by the cross‐validation technique using the R functions *perf()* and *tune.splsda()* (10‐fold, 100‐repeat as recommended) in the same package. The number of components and variables resulting in the lowest balanced error rate was selected. The optimized parameters were used to construct the final sPLS‐DA model.

To determine the significance of difference between sample types, a permutation significance test based on the cross‐validation technique was performed on the finalized sPLS‐DA model, using the function *pairwise.MVA.test()* with 1000 permutations in the R package “RVAideMemoire” (Hervé & Hervé, 2023). The default false discovery rate method was used to adjust *p*‐values in pairwise comparisons.

Three types of plots were generated based on the finalized sPLS‐DA model. First, we created a sample plot showing the ordination of sample type (small intestine, large intestine, and feces) clustering using the R function *plotIndiv()*. Second, we created a clustered image map to visualize the similarity between sample types by showing ASV associations using R function *cim()*. Third, we created loading plots showing the best characterized ASVs for the first three components in the finalized sPLS‐DA model using R function *plotLoadings()*. All functions used are available in the R package “mixOmics” (Lê Cao et al., 2016; Rohart et al., 2017).

### 2.14 Univariate differential abundance analysis

Univariate differential abundance between sample types was tested at six taxonomic levels (phylum, class, order, family, genus, and species). We performed a compositional data analysis approach by the R package “ALDEx2” (Fernandes et al., 2013, 2014; Gloor et al., 2016). The algorithm significantly reduces the problem of false‐positive identification (Thorsen et al., 2016) and works effectively for datasets of 16S rRNA high‐throughput sequencing (Bian et al., 2017). We used the R function *aldex.clr()* with 1000 Monte Carlo replicates drawn from a Dirichlet distribution and then the function *aldex.glm()* to fit generalized linear models, using the same model formula as in Shannon diversity estimates. Finally, we used the function *aldex.glm.effect()* to calculate the effect size of each taxon. To identify taxa that differ significantly between groups, we used adjusted *p*‐value (Holm–Bonferroni Family Wide Error Rate correction as recommended) and effect size estimates (cut‐off = 1) (Fernandes et al., 2013; Halsey et al., 2015).

## 3 RESULTS

A total of 154 samples were collected (60 small intestine, 60 large intestine, and 34 feces). After prefiltering, 151 samples were retained for analyses (58 small intestine, 59 large intestine, and 34 feces) (Table 1). A total of 1357 ASVs were used in the analysis with a total frequency of 1,204,688 after prefiltering. All negative controls were negative based on results from Qubit™ 3 Fluorometer (Thermo Fisher Scientific Inc., MA, USA), TapeStation system (Agilent Technologies), and sequencing at Macrogen (Seoul, South Korea). Raw sequence and metadata used in this study are available on an open data publishing platform, Dryad ([dataset] Lam & Fong, 2023).

**TABLE 1:** A summary of *Amolops hongkongensis* and *Quasipaa exilispinosa* collected in this study.

Species	Location	Weight (g)		Snout‐vent length (cm)		Number of individual	Number of feces collected
		Mean	Standard deviation	Mean	Standard deviation		
*Amolops hongkongensis*	Lion Rock	8.50	1.91	4.65	0.42	4	3
	Mui Tsz Lam	8.60	4.56	4.62	0.78	5	5
	Tai Mo Shan	8.14	2.19	4.66	0.44	7	4
	Tai Shui Hang	8.67	3.50	4.53	0.60	6	4
	Tai Tam	11.00	2.00	4.95	0.35	6	5
	Ting Kau	9.67	2.08	4.60	0.17	3	3
*Quasipaa exilispinosa*	Lion Rock	23.14	6.82	6.24	0.68	7	0
	Tai Mo Shan	28.00	9.21	6.58	0.71	6	5
	Tai Tam	26.50	6.19	6.48	0.44	6	2
	Tei Tong Tsai	36.43	7.11	7.24	0.54	7	2
	Ting Kau	18.50	2.12	5.40	0.71	2	1

### 3.1 Alpha diversity

**FIGURE 2:**
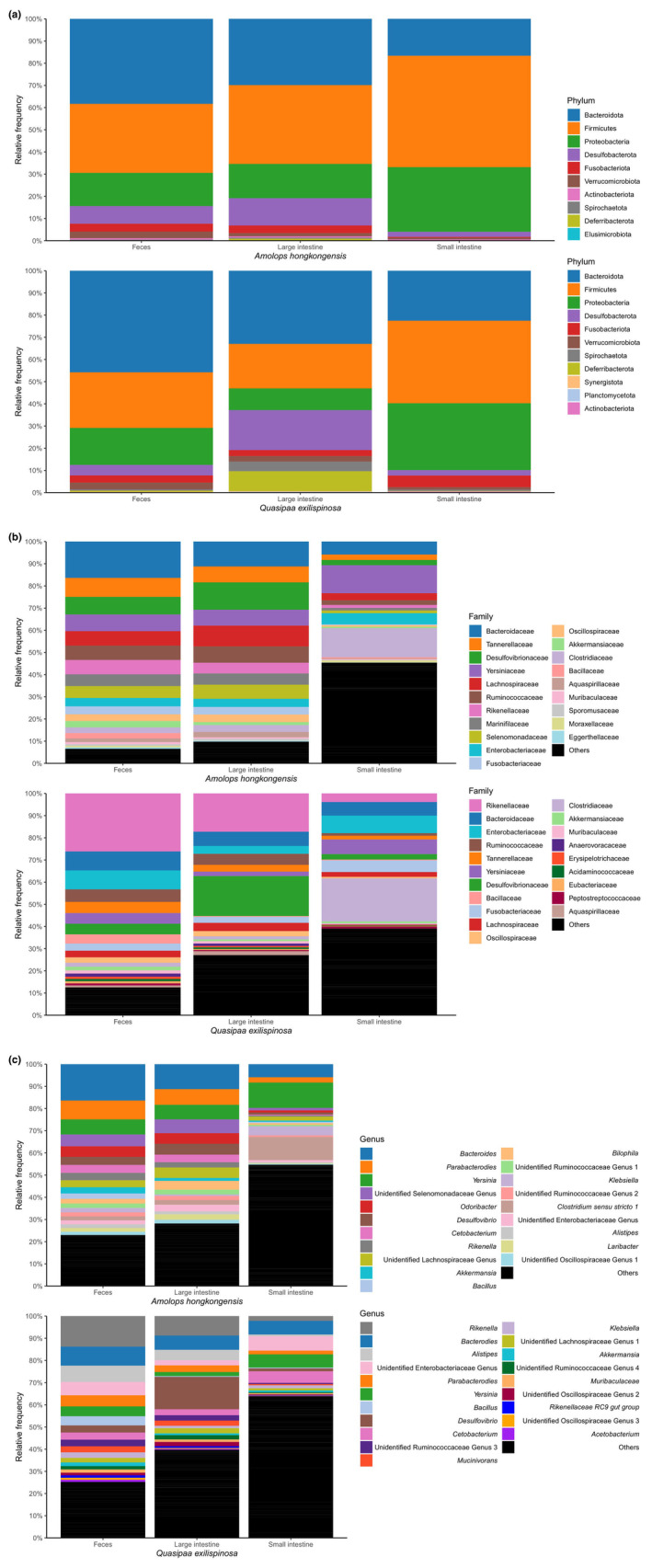
Taxa bar plot of three sample types (small intestine, large intestine, and feces) of *Amolops hongkongensis* and *Quasipaa exilispinosa* at three taxonomic levels (a) the phylum, (b) the family, and (c) the genus.

**FIGURE 3:**
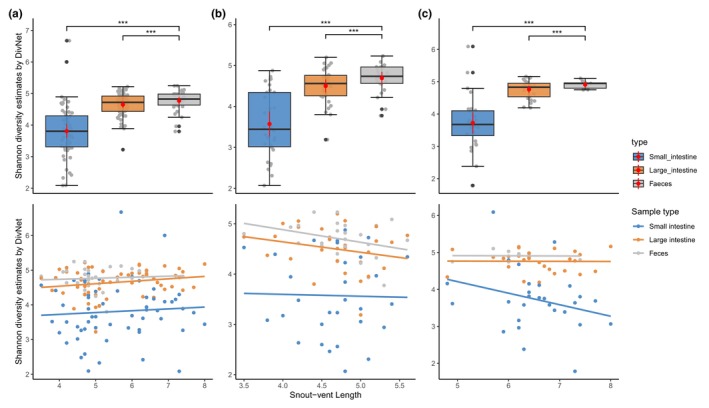
Shannon diversity estimates of three sample types (small intestine, large intestine, and feces) of (a) the combined dataset, (b) *Amolops hongkongensis* and (c) *Quasipaa exilispinosa*. Pairwise comparison with a *** indicates a statistically significant difference (*p*‐value <.01).

Microbiota of three sample types (small intestine, large intestine, and feces) at three taxonomic levels (phylum, family, and genus) separately for both species is shown in Figure 2. Both species share dominant taxa at phylum and family levels, but differ at the genus level. Comparing the presence–absence of taxa, both species share a large proportion of taxa (Figure S1). For alpha diversity, the Shannon diversity estimates were calculated at the ASV level for each species and then the combined dataset. Overall, alpha diversity of three datasets showed the same pattern—feces have the highest estimated value, followed by the large intestine, and the small intestine (Figure 3). For datasets of each species, the best model selected by AICc includes sample type and snout‐vent length as fixed factors, with individual identity as a random factor. For the combined dataset, the best model included sample type, snout‐vent length, and species as fixed factors. These best models suggest there are significant interactions (*p* < .001) between sample type and snout‐vent length (and species for a combined dataset) (Figure 3). Pairwise comparisons of Shannon diversity estimates revealed significant differences between sample type, except between the small intestine and large intestine (Table 2; Figure 3).

**TABLE 2:** Pairwise comparisons of Shannon diversity measures between feces and intestine sections (small intestine and large intestine).

Pairwise comparison	Shannon diversity estimates		
	Estimates	Standard errors	*p*‐values
Amolops hongkongensis			
Feces–Small intestine*	**−1.961**	**0.033**	**<.001**
Feces–Large intestine*	**−0.578**	**0.029**	**<.001**
Small intestine–Large intestine	1.383	1.228	.130
Quasipaa exilispinosa			
Feces–Small intestine*	**−1.007**	**0.039**	**<.001**
Feces–Large intestine*	**−0.841**	**0.042**	**<.001**
Small intestine–Large intestine	0.165	2.428	.473
Combined dataset			
Feces–Small intestine*	**−1.777**	**0.033**	**<.001**
Feces–Large intestine*	**−0.439**	**0.023**	**<.001**
Small intestine–Large intestine	1.338	1.122	.116

*Note*: Cells with bold font and * indicates a statistically significant difference.

### 3.2 Multivariate differential abundance analysis

**FIGURE 4:**
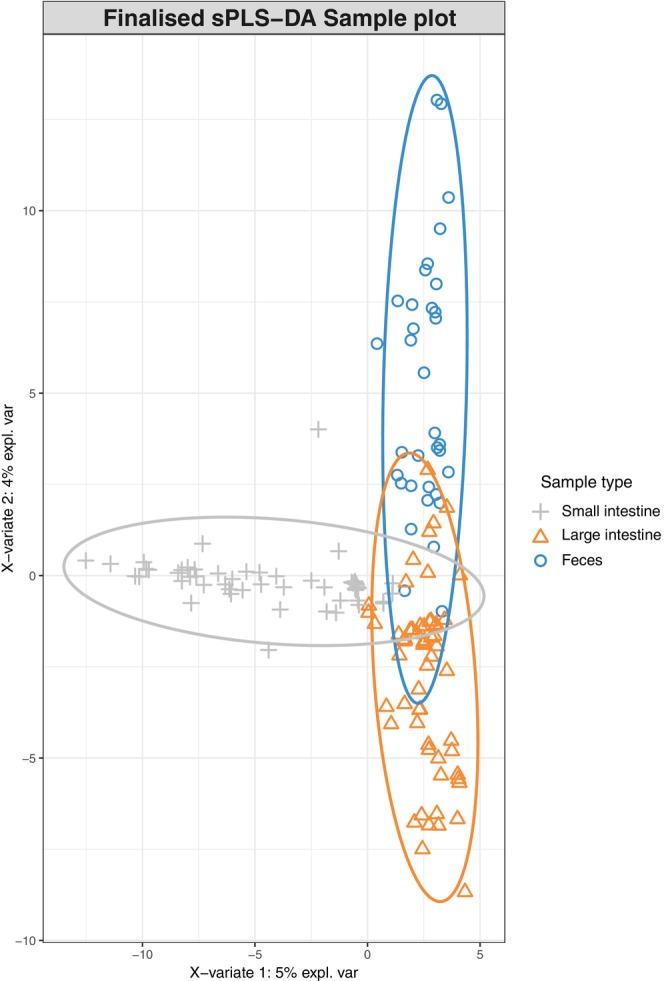
Sample plot of the finalized sPLS‐DA model based on a combined dataset.

**FIGURE 5:**
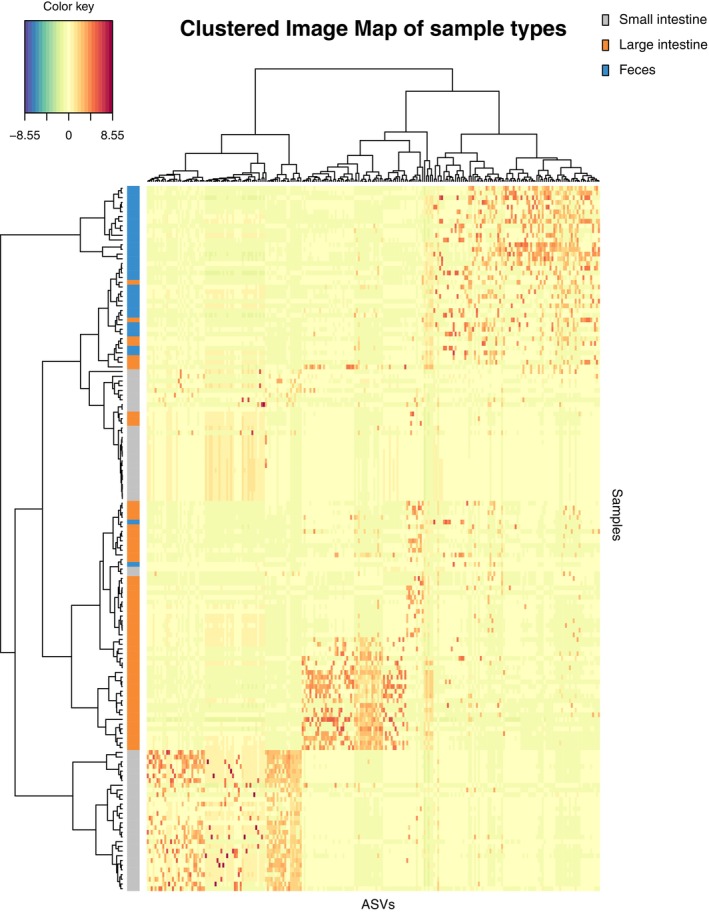
Cluster image map of the finalized sPLS‐DA model based on a combined dataset. The color key indicates the relative abundance.

**FIGURE 6:**
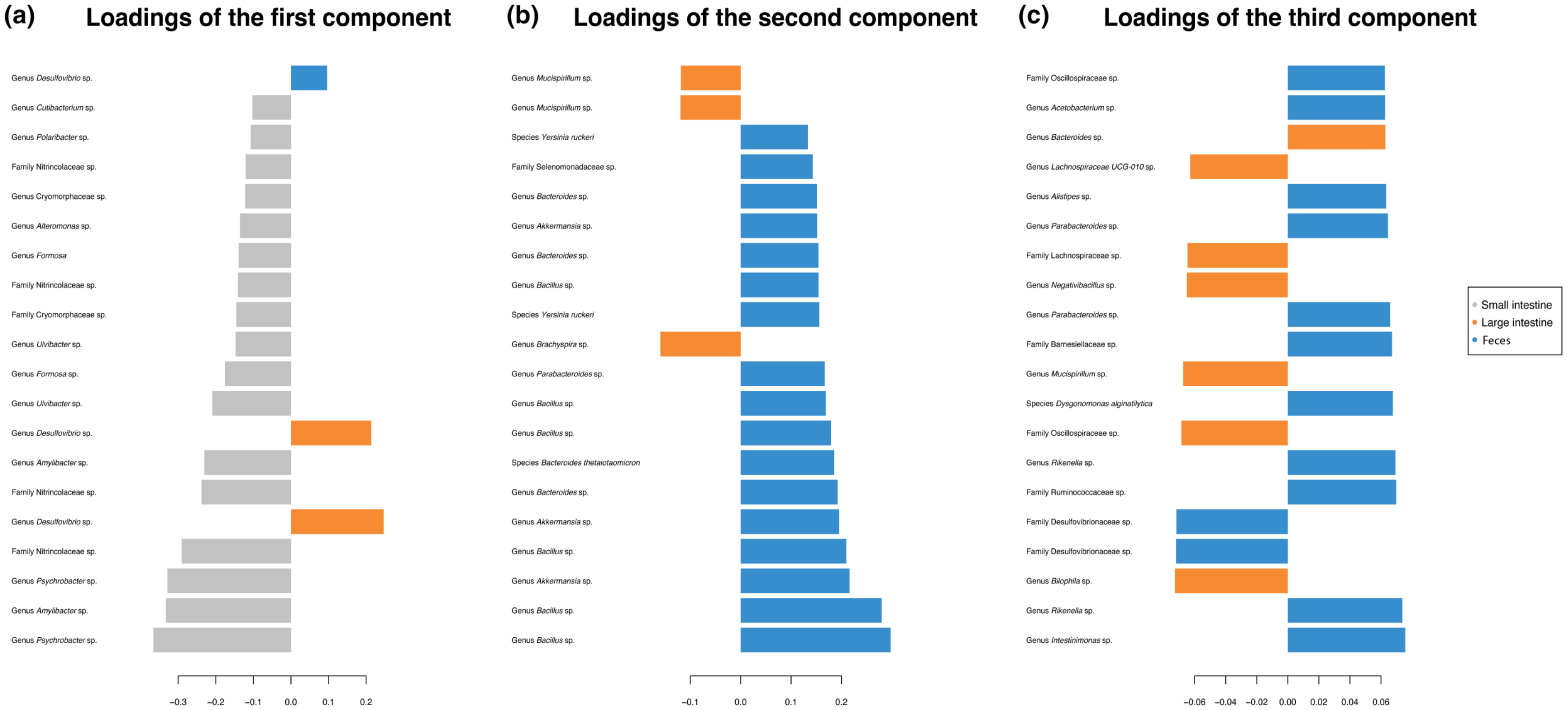
Loading plots of the first three components of the finalized sPLS‐DA model based on a combined dataset. (a) the first component, (b) the second component, and (c) the third component.

The mixMC framework was used to compare microbiota between sample types (small intestine, large intestine, and feces). The preliminary analyses conducted separately for each species recovered similar results (Figures S2 and S3), so we present the result of the analysis conducted on the combined dataset. The exploratory PCA (not shown) suggests the first two components are sufficient to finalize the model. Significant differences of microbiota between sample types were found (*p* < .001) in pairwise comparisons in the dataset for each species and the combined dataset. The sample plot using the first two components from the sPLS‐DA model shows a separation of microbiota for each sample type with minor overlap (Figure 4). Furthermore, the clustered image map shows the association between ASVs and sample type (Figure 5). Strong clusters of ASV association (Figure 5, red squares forming clusters) represent the characteristic microbiota of each sample type. Comparing the small intestine (gray bars on left) and feces (blue bars on left), overlapping of abundant ASVs is rare, suggesting distinct microbiota (Figure 5, on the left, blue bars rarely interleave with gray bars). Comparing the large intestine (orange bars) and feces (blue bars), there is more overlapping of abundant ASVs than between small intestine and feces, suggesting more similar microbiota (Figure 5, on the left, some blue bars interleave with orange bars). To identify the ASVs that best characterize each sample type, loading plots were created using the first three components of the finalized sPLS‐DA model (Figure 6). The first component selects ASVs mostly associated with small intestine samples (Figure 6a), while the second component represents ASVs mostly associated with feces (Figure 6b), and the third component shows ASVs associated with both large intestine and fecal samples (Figure 6c). The length of the bar indicates the strength of the association between the taxa and the corresponding component; taxa with longer bars indicates a stronger influence on the component. The direction of the bars indicates the direction of taxa separating the difference between sample types. The magnitude of influence of each taxon is arranged in ascending order; taxa at the bottom have the greatest influence. Only top 20 taxa with greatest influence were shown in each component.

### 3.3 Univariate differential abundance analysis

To identify ASVs that are significantly different between sample types, we performed ALDEx2 at six taxonomic levels (phylum, class, order, family, genus, and species). Using criteria of adjusted *p* < .05 and effect size >1, 33 taxa (one class, five orders, 11 families, nine genera, and seven species) are identified as having significantly different abundance between feces and the small intestine, while three taxa (one order, one family, and one genus) are identified between the feces and large intestine, and 38 taxa (two classes, six orders, 11 families,10 genera, and nine species) are identified between the small and large intestine (Table 3).

**TABLE 3:** List of taxa significantly different between sample types (feces–small intestine, feces–large intestine, and large intestine–small intestine).

Taxon	Taxonomic level	Effect size	Adjust *p*‐value
Feces–small intestine			
Alphaproteobacteria	Class	1.00	3.02E−08
Bacteroidales	Order	−1.38	1.88E−06
Oscillospirales		−1.20	3.18E−07
Desulfovibrionales		−1.18	1.49E−06
Rhodobacterales		1.29	1.76E−07
Flavobacteriales		1.48	2.91E−10
Oscillospiraceae	Family	−1.48	1.05E−08
Rikenellaceae		−1.29	3.34E−08
Tannerellaceae		−1.23	7.53E−08
Desulfovibrionaceae		−1.14	1.13E−06
Ruminococcaceae		−1.13	1.70E−06
Marinifilaceae		−1.07	1.91E−05
Cryomorphaceae		1.06	3.26E−04
Alteromonadaceae		1.12	2.14E−05
Rhodobacteraceae		1.30	5.44E−07
Nitrincolaceae		1.32	6.44E−07
Flavobacteriaceae		1.35	3.49E−08
*Rikenella*	Genus	−1.35	3.12E−08
*Desulfovibrio*		−1.31	1.09E−07
*Alistipes*		−1.29	2.58E−07
*Parabacteroides*		−1.25	9.95E−08
Uncultured Ruminococcaceae		−1.03	2.88E−04
*Alteromonas*		1.10	5.77E−05
*Amylibacter*		1.21	6.97E−06
Uncultured Nitrincolaceae		1.26	1.86E−06
*Psychrobacter*		1.30	6.22E−07
Uncultured *Rikenella*	Species	−1.14	3.20E−06
Uncultured *Rikenella*		−1.06	5.73E−05
Uncultured Ruminococcaceae		−1.04	5.06E−05
Uncultured *Alteromonas*		1.07	6.86E−05
Uncultured *Amylibacter*		1.18	1.81E−05
Uncultured Nitrincolaceae		1.21	4.34E−06
Uncultured *Psychrobacter*		1.28	6.70E−06
Feces–large intestine			
Bacillales	Order	−1.16	3.86E−07
Bacillaceae	Family	−1.11	5.98E−07
*Bacillus*	Genus	−1.08	7.16E−06
Large intestine–small intestine			
Deferribacteres	Class	−1.09	4.13E−09
Desulfovibrionia		−1.04	5.98E−12
Oscillospirales	Order	−1.39	1.45E−16
Deferribacterales		−1.34	1.14E−11
Desulfovibrionales		−1.31	4.79E−14
Flavobacteriales		1.25	1.15E−12
Rhodobacterales		1.33	2.25E−11
Pseudomonadales		1.48	4.01E−14
Oscillospiraceae	Family	−1.50	4.49E−14
Deferribacteraceae		−1.37	2.59E−11
Ruminococcaceae		−1.31	3.62E−16
Desulfovibrionaceae		−1.31	3.66E−15
Rikenellaceae		−1.24	1.00E−11
Cryomorphaceae		1.04	9.62E−08
Alteromonadaceae		1.16	1.01E−09
Moraxellaceae		1.26	3.22E−10
Nitrincolaceae		1.30	6.10E−11
Rhodobacteraceae		1.31	7.87E−09
Flavobacteriaceae		1.35	9.37E−13
*Desulfovibrio*	Genus	−1.44	3.64E−17
*Mucispirillum*		−1.37	5.15E−12
*Rikenella*		−1.24	1.38E−09
Uncultured Ruminococcaceae		−1.09	1.32E−09
Uncultured Desulfovibrionaceae		−1.04	6.51E−08
*Odoribacter*		−1.02	1.79E−09
*Alteromonas*		1.10	1.82E−08
*Amylibacter*		1.19	3.97E−08
Uncultured *Nitrincolaceae*		1.27	6.44E−08
*Psychrobacter*		1.30	1.79E−10
Uncultured *Mucispirillum*	Species	−1.37	3.74E−12
Uncultured *Rikenella*		−1.14	2.98E−09
Uncultured *Desulfovibrio*		−1.11	1.75E−08
Uncultured Desulfovibrionaceae		−1.08	2.22E−07
Uncultured Ruminococcaceae		−1.08	1.79E−09
Uncultured *Alteromonas*		1.07	3.59E−07
Uncultured *Amylibacter*		1.17	1.08E−07
Uncultured Nitrincolaceae		1.22	3.19E−09
Uncultured *Psychrobacter*		1.28	5.12E−10

*Note*: The positive and negative effect size indicate higher abundance in the former and latter group, respectively.

## 4 DISCUSSION

In this study, we characterized and compared microbiota (small intestine, large intestine, and feces) of two Hong Kong stream‐dwelling frog species: Lesser Spiny Frog (*Q. exilispinosa*) and Hong Kong Cascade Frog (*A. hongkongensis*). In terms of presence–absence, both species are similar at the phylum and family levels but differ at the genus level (Figure S1). Next, we evaluated the effectiveness of using feces as a proxy of intestinal microbiota in these two amphibian species. Although cloacal swabs are also a commonly used sampling approach, in this study, we focus on feces. We found that feces are not representative of both the small and large intestine, as evidenced by a significant difference of Shannon diversity estimates (Table 2), a distinct microbiota between three sample types (small intestine, large intestine, and feces) (Figures 4 and 5), and significant pairwise comparisons (Table 3). We provide a detailed explanation below.

As the subsections of the intestine have different environmental conditions that influence the microbiotas, it is infeasible for feces to mimic the microbiota of all subsections. We therefore shift the discussion to “how much overlap is there between feces and small/large intestine?” and “is the feces microbiota more similar to small or large intestine?”

### 4.1 How much overlap is there between feces and small/large intestine?

Alpha diversity provides a general view of microbiota within each sample type. The significant difference of Shannon diversity estimates between the small and large intestine indicates a change in the microbiota along the gut (Table 2). Another study of amphibian microbiota studying a terrestrial species, the Cane Toad (*Rhinella marina*) (Zhou, Nelson, et al., 2020), similarly found a significant difference between sample types (cloaca–small intestine, feces–small intestine, and large intestine–small intestine), although the highest alpha diversity was found in the large intestine (feces in our study).

Multivariate analysis further investigates the differences of microbiota between sample type. Although the sample plot suggests three sample types share a common set of microbiota, it emphasizes a high degree of difference (Figure 4). In addition, the clustered image map supports the high degree of microbial difference between sample types by showing a clear separation of clusters between feces and both intestinal sections in terms of a low number of shared taxa and low color intensity of shared taxa (Figure 5). The difference of the microbial pattern is supported by the significant results in pairwise comparisons. The microbiota difference between sample types found in our study agrees with that in Zhou, Nelson, et al. (2020), both showing a significant difference of microbiota between feces–small intestine and feces–large intestine.

Despite the differences in the habitat in this study (aquatic) and Zhou, Nelson, et al. (2020) (terrestrial), both studies have similar results in terms of alpha diversity and microbiota difference. To sum up, there is limited overlap between the microbiota of feces and small/large intestine, indicating feces is not an appropriate proxy of both intestinal sections.

### 4.2 Are feces more similar to the small or large intestine?

Next, we evaluate which section of the intestine is better reflected by the fecal microbiota using the mixMC framework. The clustered image map (Figure 4) shows more overlap of abundant ASVs of feces–large intestine than that of feces–small intestine. These results indicate that the similarity is higher between the large intestine than the small intestine. However, we also point out that the result differed for the two species, with no overlap between the large intestine and feces for *Q. exilisplinosa* and more overlap for *A. hongkongensis* (Figure S2). The difference of overlap between the microbiota of feces and both intestinal sections may be explained by the different environments of the small and large intestine. The small intestine has a more acidic environment and shorter transition time of ingested content, which selects for rapidly growing species (Donaldson et al., 2016). In contrast, the large intestine retains undigested content for longer time and favors a more diverse community of microbiota (Donaldson et al., 2016), driving microbial difference. After defecation, microbiota changes (see discussion below). A longer time gap between small intestine–defecation than that of large intestine–defecation provides a longer duration for microbial change. Lastly, we hypothesize that collecting fecal samples with minimized time gap after defecation is expected to retain more microbiota in the large intestine. Overall, our work points toward a higher possibility of feces being a proxy of the large intestine, but additional work needs to be done with other species to determine whether this trend is generalizable.

### 4.3 Potential factors altering the fecal microbiota

The difference between the microbiota of feces and intestines could happen after defecation. Studies on wildlife microbiota may be affected by a delay between defecation and sample collection. Unlike human studies, in which the fecal sample can be preserved immediately with buffer and freezing by patients themselves following the researcher's protocol, wildlife studies face an unavoidable time gap between host defecation and sample collection. Although temporary storage of fecal samples at room temperature does not dramatically alter the microbiota (Roesch et al., 2009), the chance of environmental contamination and disproportional bacterial growth increases; Menke et al. (2015) compared the microbiota change over time of fecal samples exposed to the natural environment in two ungulate species and found a large difference between species regarding the rate of microbiota change, presumably due to a mixed effect of environmental factors and fecal characteristics (Menke et al., 2015). To minimize contamination, we collected fecal samples in a sterilized environment with minimized time gap of sampling, but we cannot eliminate microbial change due to degradation. The microbiota change during fecal degradation is currently unknown in our study system. Future studies subsampling fecal microbiota over time in a controlled environment are necessary to elucidate the shift of fecal microbiota.

Currently in wildlife studies, contamination can only be minimized by rapid collection as in Yan et al. (2019), Zhou, Nelson, et al. (2020), and this study. Understanding defecation behavior of hosts may help minimize the time lag before sample collection. During a preliminary test, amphibians in captivity seemed to prefer defecating with high humidity. The effect of high humidity on the fecal microbiota is unknown and was not assessed in this study. In terms of species, *A. hongkongensis* were more likely to defecate 6–9 h after capture, while *Q. exilispinosa* were more likely to defecate after 12–18 h. With a better understanding of the defecation behavior, we believe researchers can design a schedule to increase efficiency when collecting fecal samples and minimize disturbance, which would reduce microbiota changes.

Diet is a significant contributor to fecal microbiota in a wide range of taxa, such as mammals (van Leeuwen et al., 2020; Zoelzer et al., 2021), birds (Schmiedová et al., 2022; Wang et al., 2022), fish (Härer & Rennison, 2023; Ringø et al., 2016), reptiles (Fong et al., 2020; Hoffbeck et al., 2023; Jiang et al., 2017), and amphibians (Chang et al., 2016; Wang, Smith, et al., 2021; Wang, Wang, et al., 2021). Diet can alter fecal microbiota in multiple ways. While nutrients differ in diet may be responsible for the differentiation of microbial growth (Zhang, 2022), microbiota can originate from undigested food content, leading to the difference of microbiota across diet. In wildlife diet studies, feces can be used to study diet via microscopy assessment of the undigested material and DNA metabarcoding, suggesting ingested DNA can be amplified and sequenced from feces. Therefore, we should expect a portion of microbiota found in feces to originate from the undigested content rather than the host's intestine. The different sources of microbiota thus contribute to the difference of intestinal and fecal microbiota. When it comes to the use of the fecal sample as a proxy of intestinal samples, researchers risk collecting unreliable data by mixing microbiota from host and undigested food content.

Captivity alters the intestinal and fecal microbiota in mammals (Jiang et al., 2021; Li et al., 2022; McKenzie et al., 2017; Wang, Smith, et al., 2021; Wang, Wang, et al., 2021), reptiles (Fong et al., 2020; Zhou, Zhao, et al., 2020), and amphibians (Tong et al., 2019). A variety of factors in captivity were proposed to affect host‐associated microbiota, including diet, medical interventions, reduced activity range, reduced habitat contact, reduced interaction with other species, and exposure to human‐associated microbiota (Alberdi et al., 2021; McKenzie et al., 2017; Wang, Smith, et al., 2021; Wang, Wang, et al., 2021). We expect captivity to have little to no effect in our study since individuals were kept in captivity for a relatively short time (12–24 h before dissection), and no food was provided.

To date, studies comparing fecal–intestinal microbial differences in wildlife are limited, making generalization across taxa difficult. The result of this study is in line with those of recent studies in the field, suggesting a significant difference of microbiota between feces and intestines (Griffin et al., 2021; Ingala et al., 2018; Zhou, Nelson, et al., 2020). While common factors shaping intestinal microbiota are well‐studied, including the host's ecology, diet, and physiology (Brooks et al., 2016; Rojas et al., 2021; Song et al., 2020), but factors driving the difference of fecal–intestinal microbiota remains poorly understood. Additionally, analytical methods may impact results; for instance, a non‐compositional data analysis approach may lead to spurious result (McMurdie & Holmes, 2014). To make more generalized conclusions, future studies are recommended to compare fecal–intestinal differences across multiple species with distinct ecology (i.e., different habitats) and diet.

## 5 CONCLUSION

A critical concern in intestinal microbiota studies is how well feces represent the intestinal microbiota. In our study, we found that feces fail to represent the general community structure of both intestinal sections. Hence, the sacrifice of amphibians is preferred for studies when precise data are needed for gut microbiota community. The need to sacrifice animals hinders the study of intestinal microbiota in rare and endangered, as well as small‐sized amphibians where sampling intestinal microbiota via dissection becomes more challenging. In these scenarios where the use of feces is the only practical choice, we recommend that the study clearly state that the data are of fecal samples. A list of taxa differed significantly between feces and intestine (Table 3), and if future studies consistently identify the same taxa to differ, it may be possible to adjust and correct for these differences. While the use of feces may be subject to bias induced from contamination and captivity, future laboratory studies may help elucidating if there are any potential effects. Overall, each sampling method has its strengths and weakness. The trade‐off between the need of animal sacrifice and data preciseness is subject to the scope of each study. To avoid overinterpretation, the sampling method should be explicitly stated. We encourage future studies to explore and develop more precise methodologies.

## AUTHOR CONTRIBUTIONS

**Ivan P. Y. Lam:** Conceptualization (equal); data curation (lead); formal analysis (lead); funding acquisition (supporting); investigation (equal); methodology (lead); resources (supporting); validation (supporting); visualization (lead); writing – original draft (lead). **Jonathan J. Fong:** Conceptualization (equal); data curation (supporting); formal analysis (supporting); funding acquisition (lead); investigation (equal); methodology (equal); project administration (lead); resources (lead); supervision (lead); validation (equal); visualization (supporting); writing – review and editing (lead).

## CONFLICT OF INTEREST STATEMENT

The authors declare that there are no conflicts of interest.

## Supporting information


Figures S1–S3

## Data Availability

Genetic data: Raw sequence reads are deposited in Dryad. Sample metadata: Metadata are deposited in Dryad. The data that support the findings of this study are openly available in Dryad at https://datadryad.org/stash/share/dMx‐cc4oBvb0JNvgeF7D2kyM6H9hbOef4XW0vgAbeVo.
