# Supplementary figures and images for: Are fecal samples an appropriate proxy for amphibian intestinal microbiota?
Source: Ecol Evol. 2024 Jan 31;14(2):e10862. doi: 10.1002/ece3.10862 (PMC10828907; doi:10.1002/ece3.10862)

Supplementary Figure 1.


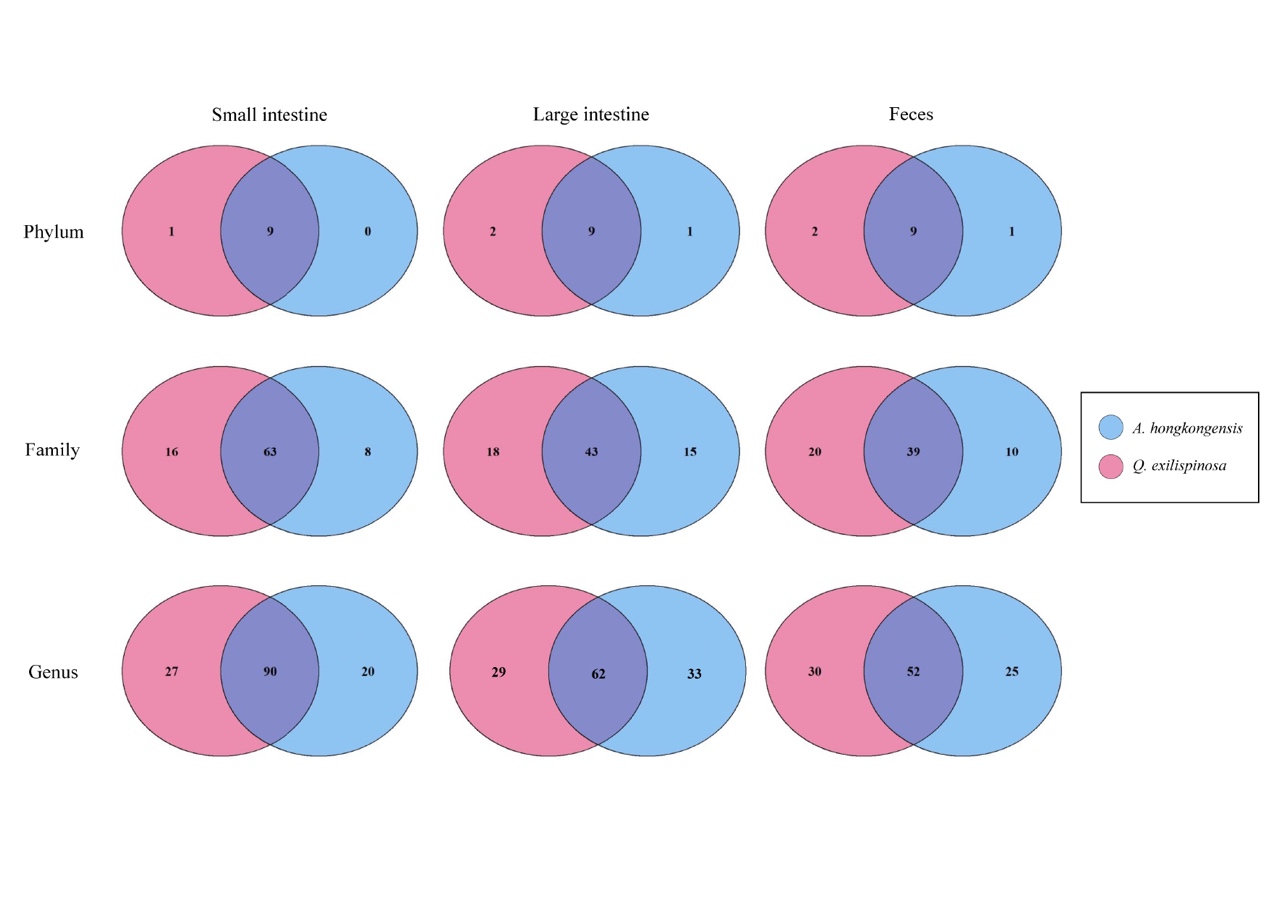


Supplementary Figure 2


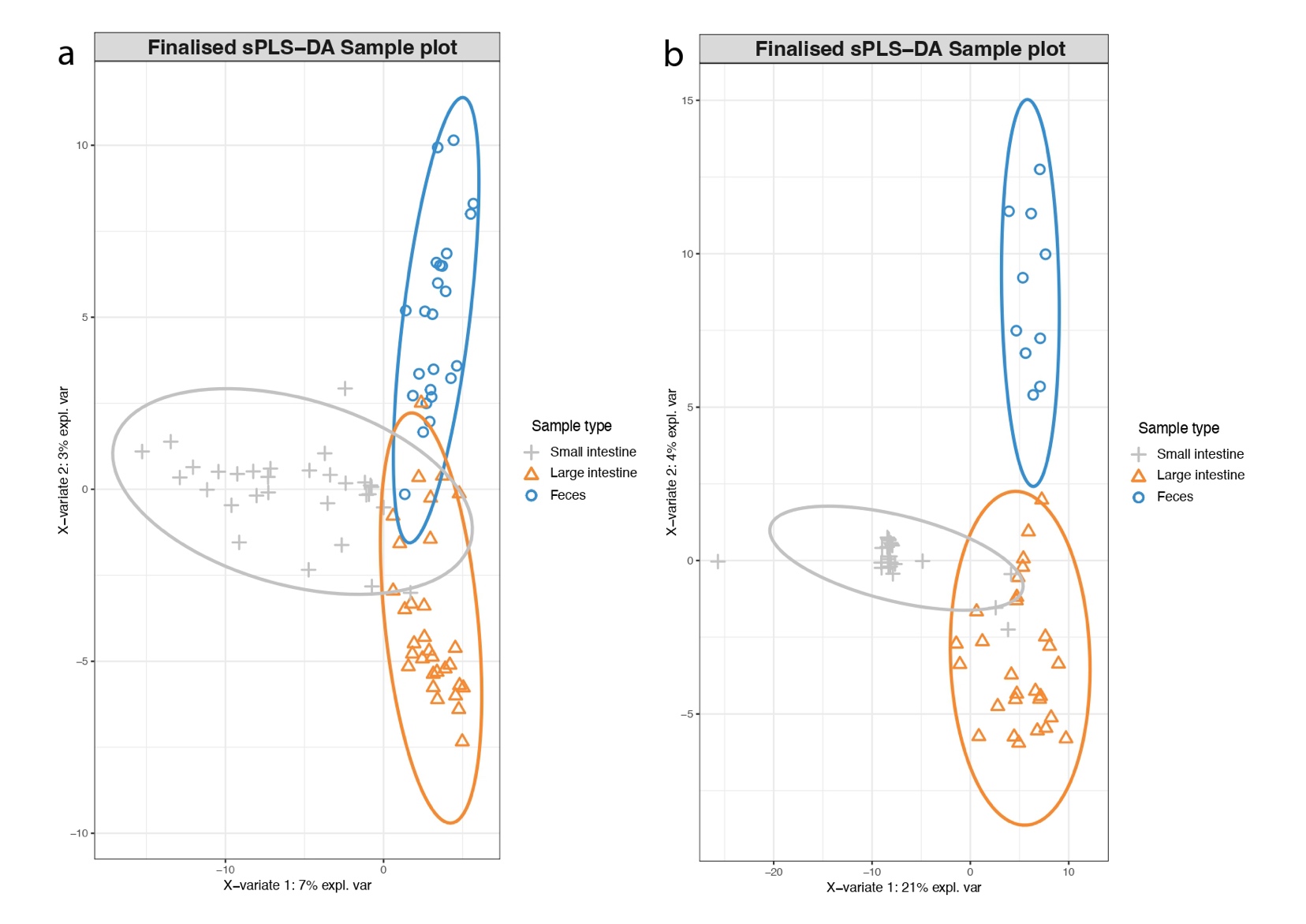


Supplementary Figure 3.


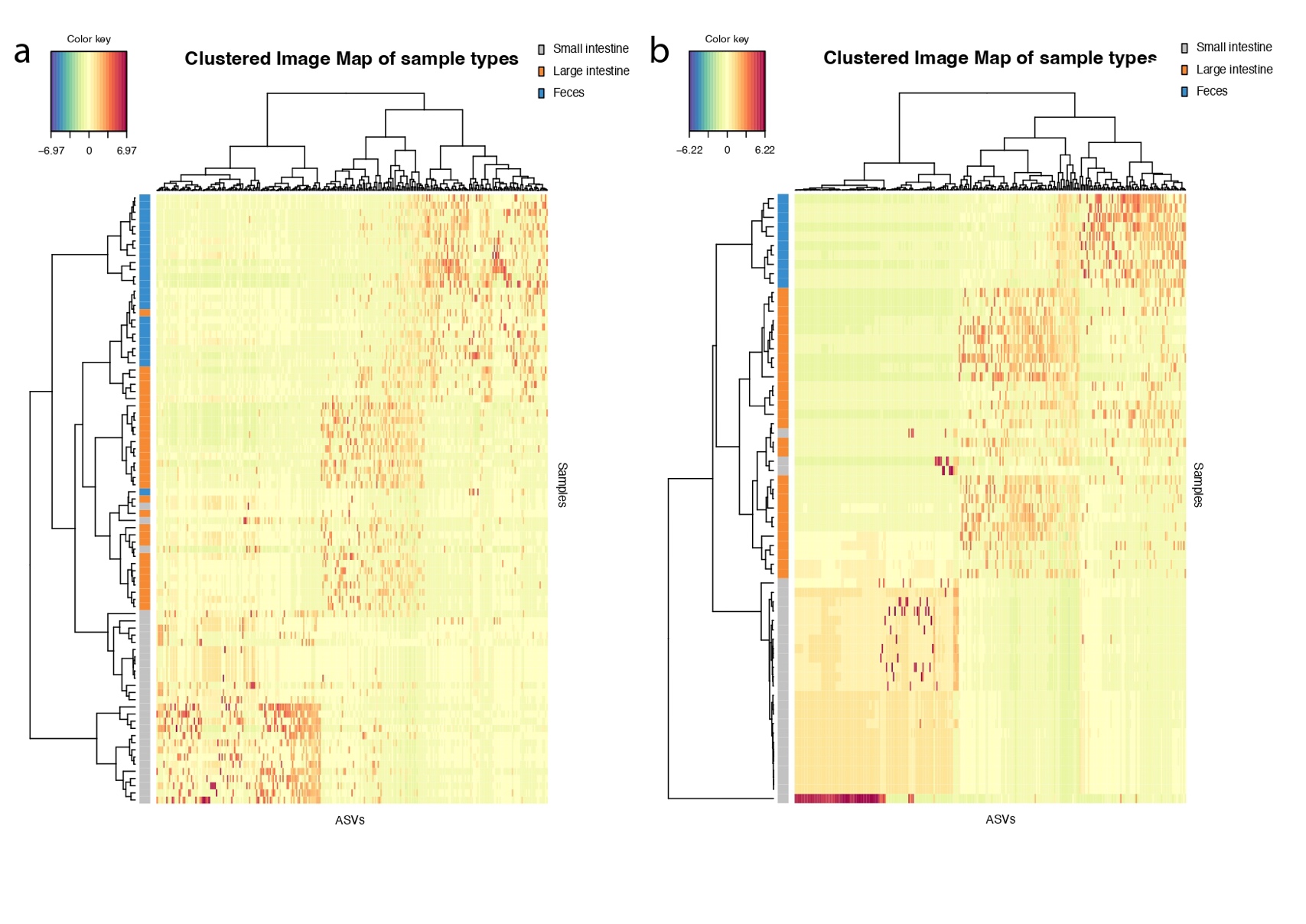

Supplement: Supplementary file 1 — Figures S1–S3 [file ECE3-14-e10862-s001.docx]
